# Are We Ready to Implement Molecular Subtyping of Bladder Cancer in Clinical Practice? Part 2: Subtypes and Divergent Differentiation

**DOI:** 10.3390/ijms23147844

**Published:** 2022-07-16

**Authors:** Francesca Sanguedolce, Magda Zanelli, Andrea Palicelli, Stefano Ascani, Maurizio Zizzo, Giorgia Cocco, Lars Björnebo, Anna Lantz, Matteo Landriscina, Vincenza Conteduca, Ugo Giovanni Falagario, Luigi Cormio, Giuseppe Carrieri

**Affiliations:** 1 Pathology Unit, Policlinico Riuniti, University of Foggia, 71122 Foggia, Italy; 2 Pathology Unit, Azienda USL-IRCCS di Reggio Emilia, 42123 Reggio Emilia, Italy; magda.zanelli@ausl.re.it (M.Z.); andrea.palicelli@ausl.re.it (A.P.); 3 Pathology Unit, Azienda Ospedaliera Santa Maria di Terni, University of Perugia, 05100 Terni, Italy; s.ascani@aospterni.it; 4 Surgical Oncology Unit, Azienda USL-IRCCS di Reggio Emilia, 42123 Reggio Emilia, Italy; maurizio.zizzo@ausl.re.it; 5 Radiotherapy Unit, Policlinico Riuniti, University of Foggia, 71122 Foggia, Italy; gcocco@ospedaliriunitifoggia.it; 6 Department of Medical Epidemiology and Biostatistics, Karolinska Institutet, 17177 Solna, Sweden; lars.bjornebo@ki.se (L.B.); anna.lantz.1@ki.se (A.L.); 7 Department of Molecular Medicine and Surgery, Karolinska Institutet, 17177 Solna, Sweden; 8 Department of Medical and Surgical Sciences, Policlinico Riuniti, University of Foggia, 71122 Foggia, Italy; matteo.landriscina@unifg.it (M.L.); vincenza.conteduca@unifg.it (V.C.); 9 Department of Urology and Renal Transplantation, Policlinico Riuniti, University of Foggia, 71122 Foggia, Italy; ugofalagario@gmail.com (U.G.F.); luigi.cormio@unifg.it (L.C.); giuseppe.carrieri@unifg.it (G.C.); 10 Department of Urology, Bonomo Teaching Hospital, 76123 Andria, Italy

**Keywords:** bladder cancer, molecular classification, immunohistochemistry

## Abstract

Following several attempts to achieve a molecular stratification of bladder cancer (BC) over the last decade, a “consensus” classification has been recently developed to provide a common base for the molecular classification of bladder cancer (BC), encompassing a six-cluster scheme with distinct prognostic and predictive characteristics. In order to implement molecular subtyping (MS) as a risk stratification tool in routine practice, immunohistochemistry (IHC) has been explored as a readily accessible, relatively inexpensive, standardized surrogate method, achieving promising results in different clinical settings. The second part of this review deals with the pathological and clinical features of the molecular clusters, both in conventional and divergent urothelial carcinoma, with a focus on the role of IHC-based subtyping.

## 1. Introduction

According to current guidelines [1,2,3], management of BC is largely based on clinical and pathological criteria, which proved to be inadequate to reliably predict treatment efficacy and ensuing prognosis [4]; the assessment of the molecular alterations underlying bladder carcinogenesis can be a valuable tool in risk stratification and targeted therapy [5]. Over the last decade, several attempts have been made to unveil the molecular heterogeneity of BC by using whole genome- and transcriptome-expression profiling, resulting in the identification of distinct molecular subtypes [6,7,8,9,10,11], mostly resembling the intrinsic basal and luminal subtypes identified in human breast cancers [7,8], in keeping with the expression signature of normal basal, intermediate, and luminal urothelial cell layers [12]. The high rate of overlap of these approaches led to a consensus summary published in 2020 under the supervision of the Bladder Cancer Molecular Taxonomy Group (BCMTG) [13]. According to this classification, there are six molecular classes of muscle invasive BC (MIBC), namely Luminal Papillary (LumP), Luminal Non-Specified (LumNS), Luminal Unstable (LumU), Stroma-rich, Basal/Squamous (Ba/Sq), and Neuroendocrine-like (NE-like), with Ba/Sq and LumP tumors being the most common, since they account for 35% and 24% of all MIBCs, respectively [13]. Tumors belonging to different groups display distinct features in terms of driver gene mutations, histological patterns, clinical behavior, and response to frontline chemotherapy [7,14,15].

Although some issues exist which further complicate an optimal stratification of BC, namely intratumor heterogeneity and variant histology [16], molecular subtyping indeed carries a valuable prognostic and predictive potential; its application in the clinical practice is hampered by the limited availability of such expensive and time-consuming methods. In an effort to translate basic research findings to clinical application, several studies focused on the use of immunohistochemistry (IHC) as a reliable, relatively inexpensive, less time-consuming, and readily accessible surrogate for high-throughput technologies [17]. IHC is a well-established method to allow subtyping of breast cancer in the clinical setting (luminal A, luminal B, ERBB2-overexpression, and basal-like) according to gene expression models, by using a four-marker panel (ER, PR, HER2, and Ki-67) [18].

Therefore, using a selected panel of antibodies, an accurate BC subtyping according to the major defined grouping might be achieved by IHC as well in surgical pathology diagnosis [7,19,20]. As known markers of basal urothelial cells and stem/progenitor cells, high-molecular-weight cytokeratins (CK) 5, 5/6, and 14, and P40, P63, CD44 have been used, alternatively or combined, to identify basal-type BCs; on the other hand, luminal tumors express markers of urothelial differentiation, namely CK20, GATA3, FOXA1, and uroplakins [6,7,10,19,20,21,22]. Robust consistency has been reported between mRNA expression profiles and IHC-based typing using luminal and basal markers [19]. A more complex approach has been proposed by the Lund University Group [11,23]. They used 28 antibodies in order to define four main groups, roughly comparable to luminal, basal, mesenchymal-like, and neuroendocrine (NE)-like categories, with luminal tumors being further subclassified in urothelial-like (Uro) (P16−, RB1+, CCND1+, FGFR3+) and genomically unstable (GU) (P16+, RB1−, CCND1−, FGFR3−) types. Obviously, by applying such a wide panel of markers, most of which being not readily accessible in the clinical setting, the requirements for using IHC in the routine practice are no longer met. Attempts have been made to develop a simple IHC-based classifier for luminal and basal phenotypes in non-muscle invasive BC (NMIBC) as well, by using GATA3, CK20, ER, Uroplakin II, HER2, and CK5/6, CD44, respectively, with conflicting results so far [24].

All in all, there is an ongoing effort to characterize a limited panel of antibodies with robust prognostic and predictive potential. The aim of this review is to provide a systematic appraisal of the state of the art in this developing field, by assessing the clinical, pathological, and prognostic features of IHC-defined subtypes of BC, including both conventional urothelial carcinomas (UCs) and tumors with divergent differentiation.

## 2. IHC-Based Molecular Subtypes

The main findings from selected studies assessing the prognostic and predictive roles of IHC-defined molecular subtypes are listed in Table 1.

### 2.1. Basal-like Tumors

Basal-like, or basal/squamous cell carcinoma-like (Bas), is usually regarded as the most aggressive subtype, showing unfavorable pathological features (namely, multifocality, higher stage, and the presence of nodal metastases), along with low survival rates [8,10,20,28,36], as well as the most represented in the consensus molecular classification [13]. Due to their longer lifespan as compared to umbrella cells, basal cells are expected to collect a higher number of genomic changes, including alterations in their chromatin landscape, such as mutations in histone- and chromatin-modifying genes [8]. The Bas subgroup is less frequent than the luminal (Lum) one, as reported by several studies [13,29,37].

Bas tumors, formerly labeled as the TCGA cluster III/IV, the Lund “urothelial-like B” subtype, and the Lund “squamous cell carcinoma-like” subtype [38], show high levels of genes associated with cells present in the basal layer within normal urothelium or stem cells, resulting in their staining with basal urothelial stem cell markers (e.g., EGFR, CD44, CK5 and/or CK5/6, and CK14), while lacking markers of urothelial differentiation (e.g., luminal transcription factors GATA3 and FOXA1, and CK20) [7,8,38,39,40]. In this subtype, basal-type antibodies stain strongly and diffusely the neoplastic cells, likewise the basal-like phenotype of breast carcinomas [41], thus losing the direct contact with the epithelial-stromal interface.

Furthermore, basal tumors usually express epithelial-mesenchymal transition (EMT) markers [42], which result in neoplastic cells acquiring mesenchymal phenotypes, with increasing progression and metastatic potential [43,44]. In the study by Kim et al., CK5/6-positive tumors were significantly associated with lower PFS and enriched with the IL6-JAK-STAT3 and TNF-α/NF-κB signaling pathways [42], which are both significantly associated with higher aggressiveness and adverse clinical outcomes in various tumors [45,46]. High cell adhesion gene expression signature, especially basolateral cell adhesion-related genes, was reported by different studies [11,42]. Furthermore, Bas tumors are enriched for squamous histological features in approximately half of cases [6,7,8,10,11,13,47].

In a recent study on a cohort including both NMIBCs and MIBCs, Bernardo et al. reported that Bas tumors were diffusely CK5-positive, with a linear correlation between CK5 expression and increased keratinization; conversely, these tumors showed low to absent stain for urothelial differentiation markers, such as PPARG, GATA3, and CK20 [48]. Furthermore, Bas featured a diffuse, full-thickness, unpolarized proliferation activity, unlike urothelial-like tumors, as demonstrated by staining for CCNB1 and KI67 throughout the tumor parenchyma [22].

Interestingly, a consistent association between basal subtype and female gender has been reported [11], thus suggesting that females are more likely to develop UCs with a keratinized/squamous phenotype associated with an adverse prognosis. In the recent study by Bontoux et al., the basal subgroup identified by coordinate expression of CK5/6 and CK14 showed significant association with higher Ki67 index and advanced pT stage, with a trend to occur in younger patients (≤65 years old); no gender differences were observed [29]. Olkhov-Mitsel et al. described significantly lower rates of disease-specific survival (DSS) in their CK5/6+/GATA3- basal-like tumors, both at univariate and multivariate analysis [26]. Accordingly, CK5/6+/CK20− basal tumors showed significantly poor PFS (*p* = 0.008) in the study by Kim et al. [42].

Bas BCs have been reported to show higher sensitivity to chemotherapy as compared to Lum and P53-like tumors, yielding improved survival in patients treated with neoadjuvant chemotherapy (NAC) in comparison to radical cystectomy (RC) alone, possibly due to their higher proliferation rates [8,15,28,49,50]; nonetheless, data from the literature are sometimes discordant [7,15,50,51], and in a recent study the cluster of basal-like tumors was significantly enriched with wild-type P53 expression, suggesting lower rates of treatment response [28].

Two recent studies investigated the predictive role of Bas IHC profile in response to NAC, both reporting a significant association with complete response (*p* = 0.037 and *p* = 0.017) in tumors classified according to their CK5/6+/CK20− and CK5/6+/CK14+/FOXA1−/GATA3− expression signatures, respectively [17,31].

Since Bas tumors show high immune cell infiltration, including high levels of tumor-associated immune cells (mostly CD8+ T lymphocytes with higher inhibitory molecular expression) and tumor-associated neutrophils [21,27,52,53], and are enriched with CD274(PD-L1)-positive tumor cells, they could be good candidates to immunotherapy [10,13,21,42,54,55,56]. PD-L1 expression is associated with effective immune escape, hence it could be a further mechanism to support disease progression in this group. Accordingly, in a clinical trial using the SP142 assay, Bas tumors stratified into TCGA clusters III and IV by gene expression profiling showed high PD-L1 expression in both immune cells and tumor cells [57], in keeping with other studies [55]. Nevertheless, in their phase 2 clinical trial (IMvigor210), Mariathasan et al. [58] described low response rates to atezolizumab as second-line therapy in Bas BCs from patients previously treated with platinum-based chemotherapy.

High rates of EGFR expression, along with an activated EGFR pathway were seen in Bas BCs [22,59], and sensitivity to anti-EGFR therapy was demonstrated in cell lines and animal models in this setting [59].

### 2.2. Luminal-like Tumors

Since Lum tumors are characterized by the expression of a urothelial differentiation gene expression signature, such as uroplakins, CK20, FOXA1, and GATA3 [8,10,23], they are thought to derive from terminally differentiated superficial umbrella cells [14].

As a result, they show an aberrant immunophenotypical profile encompassing CK20 and UPK3 within the tumor parenchyma, rather than restricted to terminal cell layers, in keeping with the activation of an at least partly aberrant differentiation program [22]. Furthermore, Lum tumors are enriched for activated PPAR-γ and FGFR3 mutations [60], along with frequent copy number losses of CDKN2A [10]. However, the presence of molecular heterogeneity among the Lum subgroup might explain intrinsic differences in clinical outcome and sensitivity to immunotherapy among tumors with overlapping phenotypical features [57,61,62,63]. Tumors belonging to TCGA luminal infiltrated molecular subtype show high inflammatory and stromal signature, as well as similarities to P53-like and GU BCs, according to the MDA and Lund classification systems [64]. In keeping with that, according to the consensus classification model, the Lum subtype can be segregated into three different subgroups, namely the LumP, LumNS, LumU subtypes, with different biological features and clinical outcomes (see above) [13].

LumP tumors show genomic profile of non-invasive high-grade BCs [10,13], and the clinical outcome of Lum BCs has been reported as good in the majority of studies, whether neoadjuvant treatment is administered or not. In a recent study, the GATA3+/CK5/6− Lum group showed significant association with favorable clinical features, namely a pure urothelial subtype (*p* = 0.012) and NMI tumors (*p* = 0.05) [12]. Using a 4-antibody panel, Bontoux et al. identified a Lum subgroup of GATA3+/FOXA1+/CK5/6−/CK14− tumors with significantly lower Ki67 index and T stage, and a trend to occur in older patients [29]. Conversely, Rinaldetti et al. reported that Lum tumors were associated with the worst DSS in a cohort of MIBC patients treated with RC only, at univariate and multivariate analysis [65], and in a cohort of 133 cisplatin-resistant MIBCs, the luminal-infiltrated tumors had a significantly less favorable prognosis compared to basal ones [66].

Data from clinical trials report on the high sensitivity of these tumors to anti-PDL1 immunotherapy regardless the mutation load [57], as well as their resistance to platinum-based chemotherapy [7]. Significantly higher response rates to atezolizumab have been described in the LumNS and LumU subtypes (*p* = 0.05 and *p* = 0.0044, respectively) by Kamoun et al. [13], in keeping with a previous clinical trial [57], though data from the literature are discordant [61,63].

Interestingly, those histological variants of urothelial carcinoma carrying an aggressive clinical behavior, such as plasmacytoid, micropapillary, and nested carcinomas, which have been labeled as Lum due to their gene signature and/or immunophenotypical profile (see below) [50,67,68,69], also show low response rates to NAC [50,70], yet the high rate of FGFR3 mutations might be exploited as a target to tyrosine kinase inhibitors [71].

In a cohort of UCs of the upper urinary tract (UTUCs), CK5/6−/CD44−/CK20+ Lum tumors were significantly enriched for HER2 overexpression (*p* = 0.003), thus raising the possibility that trastuzumab might be a treatment option in this setting [72].

### 2.3. Double-Negative (DN) and Double-Positive (DP) Tumors

Double-negative (DN) and double-positive (DP) subtypes are defined by the lack or co-existence of typical luminal and basal markers, respectively, in a BC showing morphological features of urothelial differentiation [12,20,26,34,73]. It has been suggested that DN tumors might cluster into the NE-like consensus class subtype [13]. Another hypothesis is that both DP and DN tumors might be different subtypes from Lum and Bas/Sq ones, as defined by a limited number of markers, or alternatively that they may correspond to tumors which are switching from one molecular subtype to another [42].

DN and DP tumors were as many as 30% and 23%, respectively, in a cohort of carcinoma in situ (CIS) lesions stratified by their CK5/6 and CK20 expression; however, they did not show any correlation with clinical outcome [74]. In a previous study by Rebola et al., a cohort of NMIBCs assessed by CK20 and CK5/6 could not fit into the Lum and Bas subgroups due to their double positivity or negativity [33]. In a large cohort of 187 ≥ pT1 BCs, DP/DN tumors were as many as 62% of all cases [29].

The DN subtype accounted for approximately 10% of cases in the study by Bejrananda et al. and was associated with the worst 5-year overall survival (OS), with a hazard ratio as high as 3.29 [30]. Accordingly, CK5−/CK20− DN tumors were as many as 13% in the study by Al-Sharaky et al. [36]. The prevalence rates of DN tumors raised to 25.7% and 60% in two cohorts of UTUCs and NMIBCs, respectively [33,75].

In the study by Kim et al., CK5/6−/CK20− DN tumors were enriched for the highest number on Gene Ontology and Kyoto Encyclopedia of Genes and Genomes pathways with significant differences as compared to their Bas, Lum, and DP counterparts, along with low gene expression on biological signature gene cluster expression analysis. Though showing the closest features to the CK5/6−/CK20+ Lum subgroup at functional analysis, DN tumors were regarded as a unique subtype [42], with the lowest P53-like signature genes, along with down-regulated cell motility. Altogether, such features suggest that DN negative tumors carry a favorable clinical behavior.

Conversely, according to other authors, DN tumors were reportedly enriched for down regulation of claudin target genes and high expression of genes targeted by P53 [8,19]. Furthermore, in a subsequent study, an activated EMT state and increased immune infiltrate with overexpression of PD-L1 was described in the small subgroup of <10% DN-BCs [20].

In the study by Jung et al., the CK5/6low/CK20low DN group was regarded as an intermediate stage between CK/6+ basal-like and CK20+ luminal-like tumors, showing a moderate expression of cell cycle progression genes, and a higher level of a protein synthetic/metabolic signature genes than luminal-like BCs [76].

All in all, DN tumors seem to show heterogeneous features according to different studies and thus warrant further characterization [8,19,21].

In a recent study, Labban et al. classified their cohort of 64 MIBCs into Lum (GATA3+/CK5/6−/CK14−, 56.7%) and DP (GATA3+/CK5/6 and/or CK14+, 43.3%), with the former being significantly associated with a better PFS (*p* = 0.039) [77]; the authors speculated that their DP tumors could be a surrogate marker for more aggressive biology, possibly due to an undisclosed P53-like signature [78]. Conversely, the CK5/6+/GATA3+ DP subtype identified in the MIBC cohort assessed by Bejrananda et al. showed a significantly higher OS of 42.8% as compared to other subgroups [30]. In the study by Al-Sharaky et al., DP tumors showed both favorable and unfavorable clinical features (namely, high grade along with lack of muscle invasion) [36].

Interestingly, several recent studies [27,30,36,73] identified combined staining for both luminal and basal markers in a variable number of cases, ranging from 37.9% to 65.7% of their BCs (mostly MIBC). The CK5/6+/CK20+ DP subgroup identified by Kim et al. showed the strongest immune signature gene expression at transcriptional analysis, and similar features as CK5/6+/CK20- Bas tumors at Gene Ontology, Ingenuity Pathway, and Gene Set Enrichment Analysis [42].

In a cohort of high-grade papillary NMIBCs, the CK5/6high/CK20high DP cases were enriched for PI3K-Akt signaling and connected to Lindgren’s cluster 1 by Gene Set Enrichment Analysis, consisting of tumors with favorable clinical outcome [76], in keeping with other studies [33,79,80].

Finally, in the study by Serag Eldien et al. [12], Lum (GATA3+/CK5/6−) and Bas tumors (GATA3−/CK5/6+) accounted for 60% and 7.5%, respectively. Furthermore, as many as 25% and 7.5% cases were DP and DN, respectively. According to the authors, the presence of the latter two groups might result from the lack of a definite cut-off in their study [34,73,81].

### 2.4. Urothelial-like (Uro) and Genomically Unstable (GU) Tumors

The top-level distinction into two subtypes, Lum and Bas, is common to all molecular classification in both NMIBC and MIBC, providing significant prognostic information [24,33,82]. Conversely, other subtyping schemes, including the Lund taxonomy and the recent consensus molecular classification, suggested that the Lum group may be further split into subgroups with peculiar biological and prognostic features [13,83,84]. Both Uro and GU express urothelial differentiation markers GATA3 and FOXA1 while lacking basal markers CK5 and CK14 [85,86], yet differ with regard to the mechanisms of cell cycle checkpoint inactivation [25]. Uro cancers inactivate the cell cycle regulator RB1 indirectly through loss of CDKN2A, which encodes the p16INK4A cyclin-dependent kinase inhibitor [87,88] and may be further split into UroA and UroB.

In the study by Bernardo et al. [48], tumors belonging to the UroB subtype were enriched for increased CK5 expression, morphological features of keratinization, and lower expression of urothelial differentiation gene signature as compared to UroA, mostly overlapping Bas tumors. Unlike Bas BCs, and similar to UroA tumors, UroB mostly show the organization of a basal cell layer, as well as a gradient of proliferation, as confirmed by a polarization of CCNB1 positive cells perpendicular to the tumor-stroma interface [48]. Interestingly, such features which allow to reliably distinguish among the UroA, UroB, and Bas subtypes are available only using an IHC-based classification model.

GU cancers inactivate RB1 directly through genomic loss and can have a worse prognosis than Uro [82,83,87,89]. GU cancers also demonstrate reduced P63 protein expression compared to Uro and have a proliferative activity as strong and diffuse as Bas tumors.

Jackson et al. reported that the GATA3+/P16−/CK5− Uro NMIBCs had earlier recurrences post-BCG treatment as compared to GATA3+/P16+/CK5− GU tumors [25]. Deep deletion or loss of chromosome 9p, including the CDKN2A locus, which encodes P16, is a recurrent alteration in early BCs [11,23,37,90], whereas GU NMIBCs do not show changes in CDKN2A nor in p16 protein expression [23,37]. This subtype is enriched for increased CD3+-infiltrated tumors, in comparison with Uro tumors, along with higher mutational burden [25,83,91], thus suggesting that poor recurrence-free survival (RFS) post-BCG treatment in the latter might be due to a weaker immune response [25]. Lack of CDKN2A/P16 alterations has been reported to portend response to BCG treatment in NMIBCs [25,91], unlike the poor prognosis described in MIBC cohorts [23,37,92], possibly due to the gradual development of further genetic aberrations in higher stage and grade tumors [25,50,83].

### 2.5. Neuroendocrine-like (NE-like) Tumors

NE-like BCs overlap with GU tumors in that both are enriched with very high proliferation rates and have similar genomic changes, mirrored by analogous cell cycle genes CDKN2A (p16), RB1, and CCND1 expression profiles [10,13,23,48]; yet, a major distinction between them is that NE-like usually lack PPARγ, GATA3, CK20, and P63 expression, as well as the Uro-diff signature, and show low ERBB2 and EGFR activity while expressing both neuroendocrine and neuronal markers, such as SOX2 and TUBB2B [10,23,37]. These tumors show aggressive clinical features with a poor outcome and account for <5% of all MIBCs [10,13,93]. Such shared features between GU and NE-like tumors, along with the frequent concurrence of the latter and conventional urothelial cancer, support the hypothesis that NE-like BCs may be clonally related to, and even regarded as a dedifferentiated stage of, GU tumors [48,94,95]. A consistent sensitivity to immune checkpoint inhibitors has been reported in NE-like tumors [13,96], as well as to etoposide-cisplatin therapy.

## 3. Assessment of UCs with Divergent Differentiation and Histological Subtypes

### 3.1. Overview

The high clinical variability of BC in terms of both disease presentation and outcome is probably due to its biological and genetic heterogeneity, underlined by the presence of divergent differentiation, such as squamous, glandular, and trophoblastic differentiation, as well as distinct histological subtypes, including micropapillary, nested and large nested, microcystic, sarcomatoid, and plasmacytoid, among others [97].

Such histological variants account for 33% of tumors retrieved at RCs, either as pure forms or mixed conventional UC or other variants [98], although their true incidence is probably underestimated due to challenges in their diagnosis. These tumors have inherent different immunophenotypical, genetic, and clinical features, which may support clinical decision-making in individual cases and thus should be described in the pathology report [99,100]. Though attempts have been made to stratify BCs with variant histology into established molecular classes, the reciprocal correspondence is overall suboptimal, probably due to their inherent genetic heterogeneity, as well as to the underrepresentation of each histological subtype in single studies (Table 2).

### 3.2. Squamous Differentiation

Squamous differentiation (SD) is most common among UCs with divergent differentiation, and it is morphologically marked by the presence of intercellular bridges and/or keratinization [97]. SD-UC should be distinguished by pure squamous cell carcinoma (SCC), which is a distinct non-urothelial histotype usually associated to a poor clinical outcome [97]. Tumors in the Bas molecular subtype according to various classifications are often enriched with squamous histology [7,27,29,31,32,36,47,101,102,106,107], and cluster together with squamous neoplasms of the lung, head, and neck according to the molecular pan-cancer analysis [10,101,111,112], though a complete biunivocal correspondence between transcriptional and morphological features does not exist [38]. According to the recent consensus classification, SD accounts for as many as 42% of all Bas tumors [13]. Recently, 22 SD-NMIBCs were classified into the Uro (8), Bas (7), GU (4), and Uro-CK5+ (3) by their staining patterns of GATA3, CK5, and P16 [25]; accordingly, signs of SD were observed in 33%, 7%, and 3% of the cases clustered in the Bas, UroB, and UroA groups, respectively [48]. In the study by Olkhov-Mitsel et al., SD-UCs yielded the highest levels of heterogeneity with 47.7% cases classified as Bas, 31.8% as Uro, and 20.5% as GU [26], in keeping with the findings from a previous study by Sjodahl et al., with most SD-UCs clustering into the SCCL (Bas) (44%) and UroB (25%) subtypes [22]. The only SD-UC case in the cohort analyzed by Labban et al. expressed both luminal and basal markers [77].

In their study reporting on the immunophenotypical profile of a series of histologic variants and their associated conventional UCs, Warrick et al. described significantly higher and lower rates of CK14 and FOXA1, respectively, in the cohort of squamous BCs, as compared to other differentiation types (namely, micropapillary, nested, and plasmacytoid) and conventional UCs [101].

On the basis of the frequent association between Bas molecular and immunophenotypical features and squamous morphology, Sjodahl et al. suggested the latter to be used to confirm the basal/SCC-like subtype even in the absence of CK14 expression [113], though squamous histological features have been reported as highly specific but poorly sensitive in identifying Bas tumors [59].

Hence, the intrinsic heterogeneity of tumors with pure or combined squamous histology needs to be specifically addressed.

### 3.3. Glandular Differentiation

Glandular differentiation (GD) is quite frequent, ranking second after SD among the forms of UC with divergent differentiation [97].

According to two studies, eight GD-UCs (7 NMIBCs/1 MIBC) and three NMIBCs were all classified into the GU subtype by their molecular and immunophenotypical signature [22,25]. A small subset of BCs with divergent differentiation (including GD) within the M.D. Anderson Cancer Center (MDACC) discovery cohort had a transcriptional signature consistent with the P53-like group [7]. Tanaka et al. examined a cohort of 118 MIBCs, including five with GD which were labeled as Uro, Bas (two cases each), and GU (one case), on the basis of the coordinated expression of CCNB1 and CK5 [35]. In keeping with this, a subset of tumors with GD showed features of Uro, GU, and Bas subtypes by a large immunohistochemical panel of antibodies [102], and the four cases of GD-UC analyzed by Ikeda et al. did not fit their IHC-based Bas/Lum classification [27]. The only case of GD-UC in the study by Jangir et al. was stained only with GATA3, thus it was classified as Lum [34]. Interestingly, low rates of GATA3 and UPKIII expression, ranging from 10% to 50%, were reported in previous studies [114,115].

The data so far seem to disclose a high degree of molecular and immunophenotypical heterogeneity in GD-UC; thus, further studies are needed on larger cohorts with strict inclusion criteria, due to the histological similarities between GD-UC and primary and secondary adenocarcinoma of the bladder.

### 3.4. Micropapillary Carcinoma

Micropapillary carcinoma (MPC) is a highly frequent histological subtype of urothelial carcinoma, featuring small clusters of neoplastic cells devoid of fibrovascular cores, often within empty, lacunar spaces, and portend an aggressive behavior with a high tendency for nodal and distant metastases [97,103]. Accordingly, as a result from a recent international collaborative multistakeholder project organized by the European Association of Urology (EAU) and European Society for Medical Oncology (ESMO), it has been recommended that T1 high-grade MPCs of the bladder should be treated with immediate RC and lymphadenectomy [116].

The vast majority of MPCs cluster into the Uro or Lum subtype, as supported by the enrichment of active PPAR, as well as the low CK20/very low CK5 profile [27,29,47,67,101,103,106,107]. The presence of divergent copy number alteration and region-specific mutations hints that MP differentiation may develop as an early event in UC tumorigenesis [47].

Guo et al. identified a P53-like subset of such Lum MPCs with activation of wild-type P53 downstream genes, portending a poorer clinical outcome; interestingly, a tendency to lower response to cisplatin-based chemotherapy was reported in the P53-like group as compared to pure Lum MPCs, although only a small number of patients could be assayed (11 versus 6, respectively), and the difference was not statistically significant [67]. In the same study, consistent staining for GATA3 and UPKII was observed, whereas CD44 and CK14 were negative [67]. However, MPCs are usually low to no responsive to cisplatin-based NAC regardless of their molecular subtype [50]. Yang et al. reported as many as 61% (34/56) and 39% (22/56) of MPCs meeting the criteria for the Lum and P53-like subtypes, respectively, the latter defined by the presence of P21 expression; the two groups failed to yield significant differences in terms of survival on a median follow-up of 15.2 months (range, 0.57–107.3) [104]. In keeping with that, a recent study by Ravanini et al. reported on the expression of luminal IHC-based markers (CK20, GATA3) in all 39 MP-BCs examined [32].

According to the recent consensus classification, the LumNS category was enriched in MP histology and was associated with older age, and the shorter median overall survival (1.8 years) among the Uro/Lum subtypes [13]. Consistently, MPCs show higher expression of luminal markers (CD24, FOXA1, GATA3, CK20, uroplakin 2) than basal markers (CD44, CK14, CK5, EGFR, P63) [101,103,105]. The common identification of HER2 amplification, activating mutation, and overexpression in MPC is a further overlapping feature between Lum BCs and MPCs [103]. Interestingly, Warrick et al. reported on the intratumoral heterogeneity of a series of MPCs molecularly stratified according to the Lund classification, with most tumors clustering into the GU and Uro classes, in decreasing order [102]. Consistently, in the study by Tanaka et al. on the predictive role of IHC-based subtyping in a cohort of MIBCs patients treated with chemoradiation therapy, the only case of MPC was classified as GU due to its high CCNB1/low CK5 profile [35].

### 3.5. Nested and Large Nested Carcinoma

Nested (NCs) and large nested carcinomas (LNCs) are two subtypes of UCs characterized by the presence of neoplastic cells deceptively lacking overt cytological atypia, which may increase at the tumor edge; they are arranged in small to large aggregates. Of them, LNC is rare, featuring well-delineated to uneven aggregates of tumor cells, devoid of inflammation and/or desmoplastic reaction [117], and often presents at an advanced stage [97], with some reports highlighting an unfavorable clinical outcome [118,119]. The frequent expression of PAX8, a non-urothelial marker, makes the diagnosis of NC even more challenging [120]. NC patients may present at more advanced stage compared to those with conventional UC [121], yet the recurrence and survival rates are not significantly different [121].

NCs and LNCs show both transcriptional and immunophenotypical features of luminal differentiation [68,106,107,108], such as the high expression of CD24, CK20, FOXA1, and GATA3 in both subtypes [68,101,118]. Furthermore, a higher FGFR3 mutation frequency was described in pure LN tumors as compared to mixed and conventional samples in a recent study [108], which is also in keeping with the LumP group identified by Kamoun et al. [13].

Staining for basal markers, such as CK5 and CK14, has been reported as mostly lacking to absent in both NCs and LNCs [68,108], and a small subset of cases showing co-expression of luminal and basal markers was labeled as UroB as per the Lund classification [108]. Conversely, a previous study using basal markers only described the occurrence of CK5 and CD44 expression in all 14 cases of small nested and microcystic BC subtypes [122].

Recently, Johnson et al. reported on the co-expression of luminal (FOXA1, GATA3) and basal (CK5/6) markers in a cohort of NCs, the latter displaying marked heterogeneity in a subset of cases [109], in keeping with the results from the study by Warrick et al. [102], consisting with the Uro subtype according to the Lund classification [23,101]. Tumors belonging to this subtype share with NCs some histological features, such as a smooth tumor–stroma interface and nuclear monomorphism, as seen in pure NCs [22].

### 3.6. Plasmacytoid Carcinoma

Plasmacytoid carcinoma (PC) is a rare, aggressive subtype of UC defined by the presence of single, discohesive malignant cells, with eccentric nuclei and abundant eosinophilic cytoplasm with occasional vacuoles, likewise plasma cells, hence the name; loss-of-function non-sense mutations of cadherin 1 (CDH1) gene encoding for the cell adhesion protein E-cadherin can be identified in the vast majority of cases, resulting in lack of staining for e-cadherin immunohistochemical antibody [10,97,123]. Its clinical outcome is usually poor, despite the administration of conventional chemotherapy, with most tumors presenting at advanced stage, sometimes with peritoneal carcinomatosis [124], and high rates of recurrence and mortality, even upon chemotherapy; nevertheless, immune checkpoint therapy, alone or in combination, has achieved promising results in this setting according to early-phase clinical trials [61].

PCs, along with MPCs and NCs, showed high CK20/low CK5 mRNA expression rates according to Eckstein et al. [106], as well as a GATA3+/CK20+/CK5/6-/P63- immunohistochemical profile [27,69,110], thus implying a high probability for these tumors to belong to the Lum subtype [107]. Accordingly, 43% (3/7) plasmacytoid/signet ring cell UCs were GATA3+/FOXA1+/CK5/6-/CK14- in the recent study by Bontoux et al., where the PC group as a whole was labeled as “not classified” [29].

Accordingly, in a recent series of 32 PV-UCs, tumors showed a significantly lower expression of basal markers (CK5/6 and P63) in comparison with conventional UC, along with high expression of luminal markers (GATA3 and CK20) [69], in keeping with previous studies [110,125]. As for MPC, the higher levels of HER2 expression and amplification in PC as compared to conventional UC further advocate for its belonging to the Lum class of BC [69].

Nevertheless, in the study by Warrick et al., half of their seven cases of PC showed high expression of both FOXA1 and CK15, thus suggesting distinct transcriptional features, not perfectly matching the Lum/Bas categorization [101]. Such heterogeneity was confirmed in a subsequent study from the same group, where PCs were classified as GU and Uro, likewise MPCs [102]. Therefore, a study aiming to assess the proper immunohistochemical-base subtyping for PCs should address these issues by assessing a wider panel of markers on a large cohort of tumors.

Interestingly, the finding of similar amounts of CD8+T-suppressor cells and PD-L1 expression on immune cells in PCs compared to conventional UCs in a recent study suggests that such patients would be good candidates for immunotherapy with checkpoint inhibitors [69], which is the focus of ongoing clinical trials, such as the ABACUS-02 trial (NCT04624399).

### 3.7. Sarcomatoid Carcinoma

The sarcomatoid carcinoma (SC) subtype exhibits distinct morphological features reminiscent of sarcomas, and it often presents in a combined form with conventional UCs of other urothelial subtypes [97].

In the study by Warrick et al. aiming to assess molecular heterogeneity across histological subtypes of BC, SC clustered mostly in the GU, Uro, and Bas groups [102,126]. In a previous study by Choi et al., Bas tumors were analyzed through molecular profiling and could be reliably classified by the expression of immunohistochemical markers, such as CK5/6 and CD44, along with lack of CK20, in keeping with other reports [34]; in the same study, Bas MIBCs were enriched with squamous and sarcomatoid features and often associated with advanced/metastatic disease at presentation [7,105]. Accordingly, in the MIBC series assessed by Ravanini et al. [32], including 17 sarcomatoid tumors, 88% (15/17) of them belonged either to the Bas (CK5+/CK20−, 67%) or to the DN (CK5−/CK20−, 33%) subtype. In keeping with that, in the recent consensus molecular classification by Kamoun et al., SCs were overrepresented within the Ba/Sq group [13], and recently it has been suggested that sarcomatoid differentiation might result for de-differentiation of a subset of progressing Bas tumors [47,126], being enriched for loss of adherence genes, including CDH1 and Claudins, and overexpression of EMT transcriptional factor SNAI2 [126].

RT-qPCR was performed to assess mRNA detection of CK5 and CK20 in a cohort of 122 MIBCs, showing a significant association between high CK5 expression and sarcomatoid differentiation [106], in keeping with the findings by Ikeda et al. [27]. Nevertheless, in a recent study, a subset of eight SCs showed high molecular heterogeneity, in that tumors were classified as Bas (3), GU (3), and Uro (2) on the basis of a 3-antibody panel encompassing CK5/6, GATA3, and p16 [26]. The sarcomatoid subset of BCs recently analyzed by Bontoux et al. was labeled as “not classified” on the basis of a panel of antibodies including luminal (GATA3, FOXA1) and basal (CK5/6, CK14) markers [29]. On the basis of the Lund classification [23], Sjodhal et al. proposed a mesenchymal-like (Mes-like) subtype featuring a distinct GATA3−/CK5−/EPCAM− immunophenotype and enriched with vimentin expression and/or sarcomatoid morphology [113].

The identification of significant immune cell infiltration in a substantial subset of SCs may suggest their sensitivity to treatment with immune checkpoint inhibitors [71,126].

### 3.8. Lymphoepithelioma-like Carcinoma

The lymphoepithelioma-like subtype of urothelial carcinoma (LELC) is rare and shows sheets and aggregates of undifferentiated cells with ill-defined cytoplasmic borders and large pleomorphic nuclei, within a dense, often obscuring infiltrate mostly featuring lymphocytes, plasma cells, and other inflammatory cells [97].

Manocha et al. showed that a small series of 14 LELC cases analyzed through the BASE47 gene set predictor had a basal-like molecular profile, including CK5, CK6, and CK14 markers [127], in keeping with its known responsiveness to chemotherapy; moreover, LELC is enriched with a high level of immune infiltration and PD-L1 expression, suggesting a probable role of immunotherapy in this setting [127].

So far, no attempts to carry out an immunophenotypical profiling of LELC have been performed, likely because of its rarity. However, previous studies reported on the lower to absent expression of luminal markers (GATA3, CK20), compared to the higher staining for basal antibodies (CK34, E12, P63) in these tumors, in keeping with the Bas molecular subtype [115,128].

## 4. Conclusions

The recent development of molecular subtype classification in UC provides an essential tool to improve personalized treatment strategies for these patients. Transcriptomic profiling has been extensively used in earlier studies to identify the major molecular groups, lately summarized in a consensus classification. To effectively translate the prognostic and predictive potential of such schemes in clinical practice, an IHC-based algorithm would provide many benefits, including the possibility to use archival material and to discriminate among different signals from different cells.

Since conflicting results have been published, further studies focusing on selected cohorts of UCs, namely MIBCs, NMIBCs, or single variant histology types, will be crucial for patients’ decision-making, especially in the setting of target therapies.

## Figures and Tables

**Table 1:** Findings from selected studies assessing the prognostic/predictive role of IHC-based subtypes. Bas: basal-like; CR: complete response; CRT: chemoradiation therapy; DN: double negative; DP: double positive; GU: genomically unstable; Lum: luminal-like; MIBC: muscle invasive bladder cancer; NAC: neoadjuvant chemotherapy; NMIBC: non-muscle invasive bladder cancer; OS: overall survival; Uro: urothelial-like.

Reference [n#]	Immunohistochemical Markers	Subtypes	Findings
[25]	CK5, GATA3, P16	Bas, Lum, Uro, GU	**Bas**: higher grade and stage, rapid progression to MIBC in NMIBC. **Uro**: faster recurrence than **GU**.
[17]	CK5/6, CK20	Bas, Lum, DN, DP	**Bas**: significant association with complete response to NAC.
[26]	CK5/6, GATA3, P16	Bas, Lum, Uro, GU	**Bas**: significant association with divergent differentiation, and with disease-specific death compared with **Uro** (at multivariate analysis).
[27]	CK5/6, CK14, GATA3, UPKII, CK20	Bas, Lum	**Bas**: high association with squamous differentiation and the sarcomatoid variant, and with high tumor-associated immune status; highest risk of cancer-specific mortality in combination with low tumor-associated immune status.
[28]	CK5/6, CK14, GATA3, CK20	Bas, Lum	Association with different patterns of muscularis propria invasion.
[29]	CK5/6, CK14, GATA3, FOXA1	Bas, Lum	**Bas**: more advanced disease (pT3-4) vs. **Lum** (pT1-2).
[30]	CK5/6, GARA3	Bas, Lum, DN, DP	**DN**: worst 5-year OS.
[31]	CK5/6, CK14, GATA3, FOXA1	Bas, Lum, DP	**Bas**: more likely to achieve a pathological response to NAC.
[32]	CK5, GATA3, CK20	Bas, Lum, DN, DP	No significant differences in survival among subtypes.
[33]	CK5/6, CK20	Bas, Lum, DN, DP	**Lum**: independent predictor of more aggressive disease in NMIBC.
[34]	CK5/6, CK14, GATA3, CK20	Bas, Lum	Significant association with worse (**Bas**, CK14+) and better (**Lum**, GATA3+) OS, respectively.
[35]	CK5, CCNB1	Bas, Uro, GU	**GU** and **Bas**: significant independent predictors of clinical CR after CRT.

**Table 2:** IHC-based subtyping of urothelial carcinomas with divergent differentiation or belonging to distinct histological subtypes. IHC: immunohistochemistry; LumNS: luminal non-specified; LumP: luminal papillary.

Reference [n#]	Divergent Differentiation/Histological Subtype	IHC-Based Molecular Cluster
[13,22,25,26,48,101]	Squamous	Mostly basal
[22,25,27,35,102]	Glandular	Variable
[13,32,67,101,103,104,105]	Micropapillary	Mostly luminal (LumNS)
[13,68,106,107,108,109]	Nested and large nested	Mostly luminal (LumP)
[27,29,69,101,107,110]	Plasmacytoid	Mostly luminal
[23,26,29,32,34]	Sarcomatoid	Variable
